# Effect of an endodontic e-learning application on students’ performance during their first root canal treatment on real patients: a pilot study

**DOI:** 10.1186/s12909-022-03463-y

**Published:** 2022-05-23

**Authors:** Christoph Maria Färber, Martin Lemos, Sareh Said Yekta-Michael

**Affiliations:** 1 Department for Operative Dentistry, Periodontology and Preventive Dentistry, RWTH Aachen University, Pauwelsstrasse 30, 52074 Aachen, Germany; 2 Audiovisual Media Center, Medical Faculty, RWTH Aachen University, Pauwelsstraße 30, 52074 Aachen, Germany; 3 Department of Orthodontics, RWTH Aachen University, Pauwelsstrasse 30, 52074 Aachen, Germany

**Keywords:** Artificial teeth, Blended learning, COVID-19, E-learning, Endodontic teaching

## Abstract

**Background:**

E-learning has found its way into dental teaching in general and endodontic teaching in particular. The present study aimed to implement a newly developed multimedia learning application and assess its effect on students’ first root canal treatment on real patients. With the COVID-19 outbreak, the application’s performance was investigated during the pandemic.

**Methods:**

A total of 138 students in the initial clinical endodontic course participated in this study. The control group (*n* = 49) followed the traditional curriculum, including practice on artificial teeth and face-to-face teaching events. In addition to the traditional curriculum, test group 1 (*n* = 54) had access to an endodontic e-learning application containing videos demonstrating artificial teeth and patient cases. With the COVID-19 outbreak, test group 2 (*n* = 35) had no face-to-face teaching; however, endodontic patient treatments were included. The quality of students’ first root canal treatment on real patients was compared using performance and radiographic assessment items. Statistical analysis was done using Kruskal–Wallis and chi-squared tests. Test groups received a questionnaire to assess the learning application. Test group 2 also completed a COVID-19-specific survey to measure students’ perceptions of how the pandemic affected their endodontic education.

**Results:**

The results of endodontic treatments were significantly better for test group 1 (*P* < 0.001) and 2 (*P* < 0.001) than for the control group. Likewise, there were significantly fewer treatment errors in test group 1 (*P* < 0.001) and 2 (*P* < 0.001). No significant differences were found between test groups 1 and 2. Students of the test groups positively evaluated the e-learning application. Students of test group 2 expressed their fear of negative impacts on their course performance.

**Conclusion:**

The e-learning application was well-received and seemed to improve endodontic education. The results imply that the quality of education may be maintained by implementing e-learning to compensate for face-to-face teaching. As no difference was found between online and face-to-face teaching, students’ and lecturers’ concerns that endodontic education is suffering because of the pandemic may be eased.

**Supplementary Information:**

The online version contains supplementary material available at 10.1186/s12909-022-03463-y.

## Background

Performing root canal treatments requires appropriate expertise and, therefore, a certain amount of practice. Although students can perform root canal treatments in most cases with acceptable quality and adequacy [1], the error rate increases with more complex root canal systems [2]. University education can only provide theoretical and practical skills to a limited extent, but the question arises as to how education can be improved; therefore, standards of university education in endodontics have been established [3–5]. However, teachers are aware that practice is the most important factor in developing endodontic skills. One major innovation of endodontic teaching has been introducing endodontic tooth models created using cone-beam computed tomography and manufactured using 3D printing [6–9]. Based on treatment errors observed in the last five years, our department developed the DRSK RCT® endodontic tooth model, optimized due to extensive evaluations by students and instructors and proofed to contribute to endodontic teaching [10]. Attempts have been made to incorporate media into teaching effectively to enhance dental education. In blended learning, content is prepared using media, resulting in a mixed digital and analog teaching form. For example, the format of face-to-face instruction has been successfully replaced by the use of video instruction [11]. Although other studies have demonstrated that the sole use of videos instead of lectures has its limitations, the method is preferred by students and thus should play a permanent role in dental teaching [12, 13].

Flipped classroom designs are part of blended learning and provide learners with content prior to attending the lectures or seminars. These designs are another innovation in teaching that seems to be advantageous over traditional teaching methods [14, 15]. A considerable advantage is the great acceptance of this method among students [16]. Recent studies have indicated that students’ achievements can be effectively enhanced through the flipped classroom approach [14, 17].

In December 2019, the coronavirus disease (COVID-19) appeared in China and was declared a pandemic by the World Health Organization (WHO) shortly thereafter [18]. The COVID-19 pandemic brought dental education to a standstill, forcing educators to find new approaches to continue student education [19]. Due to infection control measures such as social distancing and the prescription of lockdowns, universities worldwide responded with distance learning, implementing online courses and lectures [20–22]. Medical teaching has faced the challenge of maintaining teaching events, and virtual tutor groups have constituted a promising approach to substitute for face-to-face teaching until it can happen again safely [23]. Incorporating virtual learning applications can supplant traditional teaching methods in times of the pandemic [24–26].

Dentists have an extremely high risk of infection due to aerosol-producing procedures. Moreover, most dental education consists of lectures, simulated learning, and teaching practical skills that require a significant amount of practice. Hence, dental education has significantly suffered from COVID-19 restrictions [27]. Most dental schools initially suspended practical courses, shifted to online courses, and introduced alternative performance assessments [20, 22, 28–30]. Other adaptations have included designing virtual curricula, simulation labs, distance learning, and postponing training [19]. Moreover, portable mannequins for preclinical teaching have been advocated [31].

Given the persistence of the pandemic, it is necessary to implement a long-term strategy to sustain dental education, including endodontic education [32], while improving education for the post-pandemic world [33].

Our study aimed to measure the effect of a new endodontic e-learning application associated with using an endodontic tooth model [10] to measure its effectiveness in overcoming disruptions in endodontic teaching due to the current COVID-19 pandemic. The first null hypothesis was that the newly developed e-learning application would not enhance students’ endodontic performance in their first clinical course. Secondly, it was hypothesized that during the COVID-19 pandemic, the new teaching method would not maintain the quality of endodontic teaching when face-to-face teaching was replaced entirely by distance teaching.

## Methods

**Fig. 1:**
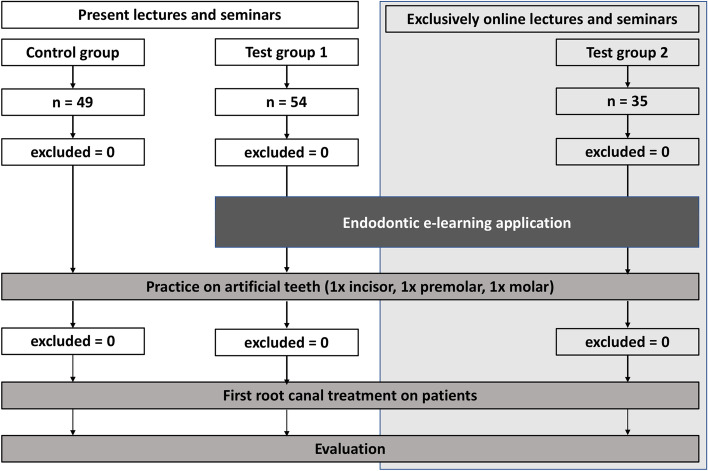
Flow diagram displaying the study design for an immediate understanding. The number of participating students and exclusions are portrayed, and the difference in received treatments is shown

The present study was conducted at the Department for Operative Dentistry, Periodontology, and Preventive Dentistry of RWTH Aachen University in Germany. After independently reviewing, the local ethics committee waived the need for ethical approval as the survey data was collected during the context of teaching. The study was initially intended to occur over four semesters. A total of 138 students were screened for participation: a control group with 49 students, test group 1 with 54 students, and test group 2 (during the pandemic) with 35 students. The number of students in each group was determined by the number of students assigned to the respective groups each semester. The authors aimed to recruit as many subjects as possible for this pilot study. The endodontic patient treatment is an examination performance of the initial clinical course. All data were collected anonymously and not included in the course evaluation. All students were examined by separate demonstrators who had no connection to the present study and no knowledge of the collected data. Participating students were assigned semester-wise into their groups for ethical reasons (Fig. 1).

Informed written consent was obtained from every participant. All participants successfully passed the preclinical endodontic course, so they already had a theoretical and practical knowledge of performing root canal treatments on extracted teeth and recently developed artificial teeth [10]. Students in the control group entered the first endodontic course without interventions other than the traditional curriculum, which contained theoretical lectures in endodontics, theoretical courses explaining the procedure of root canal treatments, exercises on extracted teeth, and an objective structured clinical examination. Before entering the clinical endodontic course, students performed practical exercises on three artificial teeth (i.e., incisor, premolar, molar). Endodontic teaching at this department was performed according to the current status [3, 4, 34]. The root canal treatment protocol contained the trepanation following primary and secondary access cavities. The negotiation of the root canal system was performed by using C-Pilot-files® (VDW®, Munich, Germany) and K-files (VDW®, Munich, Germany). After length measurement was carried out endometrically and radiographically, the root canals were enlarged with K-files up to ISO-size 25 and continued with rotary mechanical files (F 360®, Komet®, Lemgo, Germany) depending on the root canals’ initial sizes. Irrigation was performed with 3% sodium hypochlorite and 17% ethylenediaminetetraacetic acid. Eventually, the root canal system was obturated with gutta-percha points with a taper of 0.2 (Antaeos®, VDW®, Munich, Germany) using cold lateral compaction.

Test group 1 had access to educational videos incorporated in a multimedia e-learning application (AVMZ, RWTH Aachen University®) with the traditional curriculum, comprised of 48 h of practical hands-on training on extracted teeth, 7 h of theoretical seminars, and 16 h of lectures. Students could voluntarily practice further hands-on training on their own. Thus, together with the established use of artificial teeth, students could learn at their own pace and practice as often as necessary [35].

Like test group 1, the COVID-19 test group 2 experienced the traditional curriculum (exercises on extracted teeth, theoretical lectures, hands-on training on artificial teeth) and used the e-learning application. However, the lectures and seminars appeared online. Only patient treatments remained in person, performed under strict hygiene regulations.

### The multimedia learning application

**Fig. 2:**
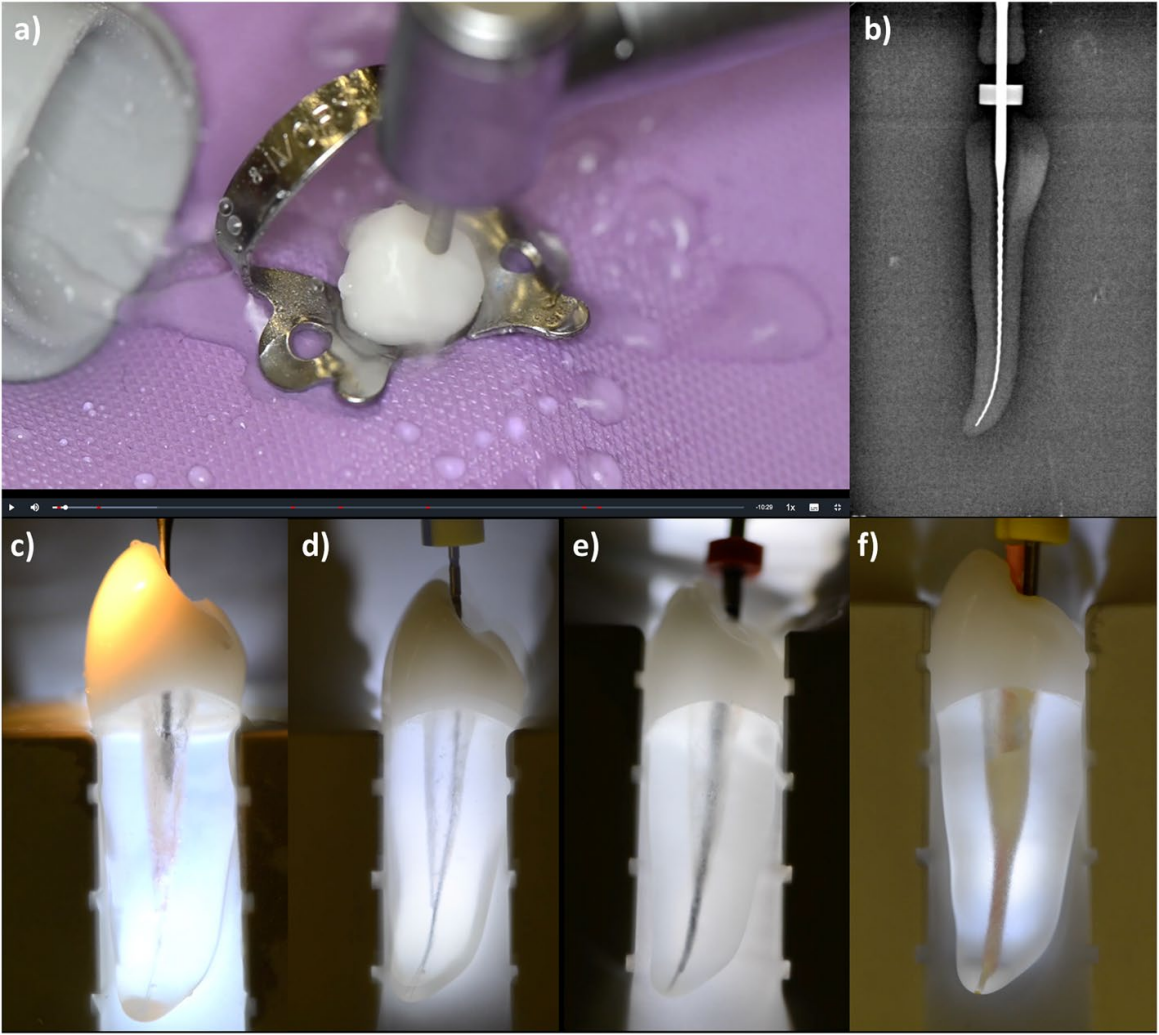
Screenshots from the endodontic e-learning application showing several steps of the routine root canal treatment on a premolar endodontic tooth model (RCT, DRSK® AB Group, Sweden): a) preparation of the access cavity under rubber dam isolation; b) radiographic length measurement using an ISO 15 K-file; c) enlarging the coronal third of the root canal system; d) sounding of the root canal system using an ISO 06 C-Pilot file®; e) flaring of the root canal walls with a mechanical file system (Reciproc®, VDW, Germany); f) obturation using the cold lateral compaction technique

The multimedia application was launched by the Audiovisual Media Centre of the Department of Medicine, RWTH Aachen University® (AVMZ), available only to the participating students of the test groups. A total of 6 educational videos were produced, showing root canal treatments according to the clinical standards of our department. An instructional video was produced for every type of tooth represented by the DRSK® RCT models (i.e., incisor, premolar, molar). Root canal treatments were demonstrated according to the American Association of Endodontists® guidelines and the current literature [3, 5, 36–38]. Through the tooth models’ translucent roots, the performed procedures were visible to the students, helping them understand the impact of each step in the process (Fig. 2).

Additionally, three videos containing a representative patient case were made available with the same procedures on real patients. The videos were filmed using a dental camera with autofocus (Elio® HD60, EKLER®, Fr) and an action camera (Hero7® Black, GoPro® Inc., US). Ambient sound was recorded with an MP3/Wave Handy Recorder (H1n, Zoom® North America, US). The videos were shortened to a maximum length of 10 min. Playability was optimized using jump tags to select specific sections of the videos. Explanatory commentary for each video was separately recorded by a tutor in the recording studio and later added to the videos. Eventually, the videos were incorporated into an online multimedia learning application accessible to students in test groups 1 and 2 via the internet and password authorization. An internet platform (https://emedia-medizin.rwth-aachen.de) widely used in medical teaching at RWTH Aachen University® was utilized. The platform is run by the AVMZ and contains various multimedia applications for several medical and dental departments. All participating students of both test groups confirmed their use of the endodontic e-learning application. The evaluation of the e-learning application assured that students had attended the online course and watched the videos.

### Endodontic teaching during the COVID-19 pandemic

At the beginning of the pandemic, our faculty, similar to almost all dental schools globally, discontinued in-person teaching and switched to online courses [28]. Instead of continuing dental teaching solely virtually and using mannequins [27, 39], a hygiene protocol was developed that made it possible to restart clinical teaching. Thus, any student could perform an endodontic treatment on a real patient.

Hygiene, distancing regulations, and drastically increased infection protection measures affected clinical teaching during the pandemic [27, 32]. Lectures and seminars were held entirely online using video communication software (Zoom®, Zoom Video Communication ®, US) to prevent unnecessary contact between individuals.

### Evaluation of students’ first root canal treatment on real patients

As the present study aimed to analyze the performance of the students’ first root canal treatment on patients, the endodontic performance during the seventh semester endodontic course was evaluated by independent demonstrators of our department. For all groups, the same demonstrators were involved. All were general practitioners with a minimum of two years of experience in endodontics. The evaluation of the treatment performance for this study was performed independently from the course assessment. The demonstrators associated with this study were not part of the first clinical course so that the result of the evaluation for this study would not conflict with the students passing the course. The evaluation sheet used in this study was designed similar to a study that examined students’ endodontic performances through items [40], resembling the evaluation sheets used in our department ‘s preclinical endodontic courses. The evaluation sheet in this study contained 21 items ranging from assessing performing straight-line access to evaluating a completed root canal filling. Notably, a maximum of 20 error points could be registered. All steps performed on the patient and the radiographs taken were evaluated because the independent demonstrators were present during treatments.

Criteria for the teeth considered for the study were freedom from pain and difficulty level suitable for the first clinical course. Molars with a moderate root canal curvature were included due to organizational reasons. For example, teeth with pronounced curvature and complex molars were excluded before the patient pool was allocated to the first clinical endodontic course since these teeth were unsuitable for students in the seventh semester performing their first root canal treatment on real patients [41]. A dentist assessed all teeth at the department before being assigned to the endodontic course.

### Evaluation of the multimedia learning application

The students in test groups 1 and 2 evaluated the learning application anonymously online using a 5-point Likert scale (1 = fully disagree, 5 = fully agree), as routinely used in medical questionnaires [42–44]. An incorporated system usability scale (SUS) was used to assess the learning application. The tooth models were rated using the same questionnaire based on a 7-point Likert scale similar to a previous study.

Finally, the COVID-19 test group answered an additional 5-point Likert scale questionnaire with 10 questions regarding their perceptions and concerns about the COVID-19 pandemic. There was no evaluation of the traditional curriculum in any of the three groups.

## Statistical analysis

All collected data were recorded in Excel® using version 16.43. Statistical analysis was performed using SPSS® (IBM® 27.0). Statistical graphics were created with either Excel® or SPSS®. The performances of the three cohorts – control group, test group 1, and test group 2 (COVID-19) – including their success and error rates were statistically analyzed using a Kruskal–Wallis test after a Shapiro–Wilk test indicated a non-normal distribution. Bonferroni correction was used as a post-hoc test. The individual items and errors between groups (dichotomous data) were statistically compared using a chi-squared and Fisher’s exact test as appropriate. For all statistical analyses, *P* ≤ 0.05 was considered significant.

The results of the Likert-scale questionnaires of test groups 1 and 2 were analyzed descriptively. A score was estimated for each participant, and an overall mean with the standard deviation was calculated. The reliability of the Likert scale was confirmed using Cronbach’s alpha.

## Results

### Treatment performance

Altogether, 138 students participated in this study. There were no dropouts regarding the evaluation of root canal treatments registered. In total, 138 teeth with 217 root canals were treated (Table 1).

**Table 1:** Number of teeth and root canals treated by students during their first real patient treatments in the first clinical endodontic course (*n* = 138)

	Incisors	Premolars	Molars
	Number of teeth (root canals) percentage	Number of teeth (root canals) percentage	Number of teeth (root canals) percentage
Control group (*n* = 49)	16 (16) 32%	23 (33) 47%	10 (34) 20%
Test group 1 (*n* = 54)	19 (19) 35%	25 (30) 46%	10 (32) 18%
Test group 2 (*n* = 35)	11 (11) 31%	17 (22) 49%	7 (20) 20%

**Fig. 3:**
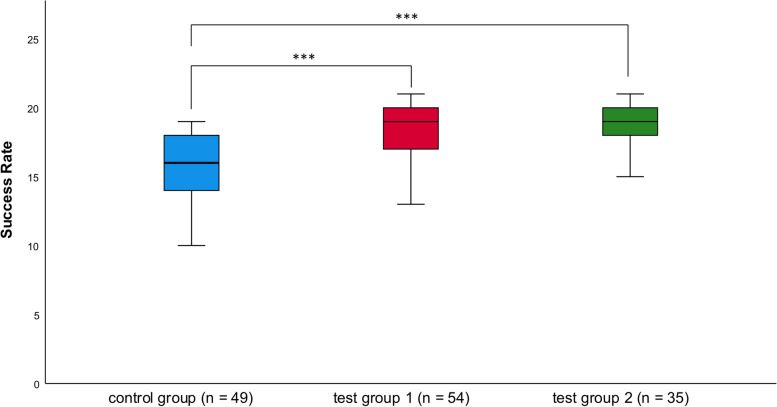
Graphic of the results of the students’ first root canal treatments on real patients during their first clinical endodontic course. The scale of the *y*-axis ranges from 0 to 21. Medians and quartiles represent the results of the three groups**.** Asterisks mark the level of significance (* for *P* < 0.05; *** for *P* < 0.001)

After implementing the endodontic multimedia application combined with additional exercises on endodontic tooth models before the first clinical endodontic course, the score of the treatment performance of the control group was 15.0 ± 3.7 (mean ± *SD*). The scores of test groups 1 and 2 were significantly higher (18.2 ± 2.2 and 19.0 ± 1.7, respectively; *P* < 0.001). There was no significant difference in treatment success in real patients between test group 1 (who experienced face-to-face teaching with access to the e-learning application) and test group 2 (who experienced exclusively digital teaching; Fig. 3).

The items that were used to assess students’ root canal treatments were divided into 4 stages.

Stage 1 contained items regarding the isolation of the tooth and the opening of the cavity. Test group 1 was significantly better at establishing the tension-free positioning of endodontic files by sufficiently removing orifices and parts of the pulp chamber roof than the control group (Item 3; *P* < 0.05). They also performed significantly better at the accurate preparation of the orifices in relation the root canal anatomy (*P* < 0.01). Stage 2 consisted of items regarding the determination and the control of the working length. Test group 1 was significantly better at the selection of sufficient working lengths according to the preoperative radiographs (Item 6; *P* < 0.01) and the adequate determination of the working lengths (Item 9; *P* < 0.05). Moreover, they more frequently documented the adequate working lengths and the reference points correctly (Item 10; *P* < 0.001). In stage 3, items for the assessment of canal preparation and irrigation were combined. Students in test group 1 were more successful in establishing tug-back with the master cone (Item 12; *P* < 0.01) and significantly better at performing radiographic controls of the fitting of their selected master cone (Item 13; *P* < 0.05). Items regarding the obturation and restoration were allocated to stage 4. When performing obturation, they shortened the root filling to a crestal level more thoroughly (Item 15; *P* < 0.01). Moreover, they cleaned the pulp chamber walls more efficiently from the rest of the sealer and gutta-percha (Item 16; *P* < 0.001). According to the post-operative radiographs of the test groups’ root canal fillings, they managed to perform better in terms of density (Item 19; *P* < 0.05), continuity (Item 20; *P* < 0.001), and extension concerning the original shape of the root canals (Item 21; *P* < 0.01).

The performance of test group 2, establishing the correct working lengths according to the preoperative radiographs, was significantly lower than test group 1 (Item 6; *P* < 0.01). However, significantly more students in test group 2 managed to achieve the adequate lengths of their root filings than in test group 1 (0.5–1.0 mm from the radiographic apex; Item 17; *P* < 0.05).

### Treatment errors

**Fig. 4:**
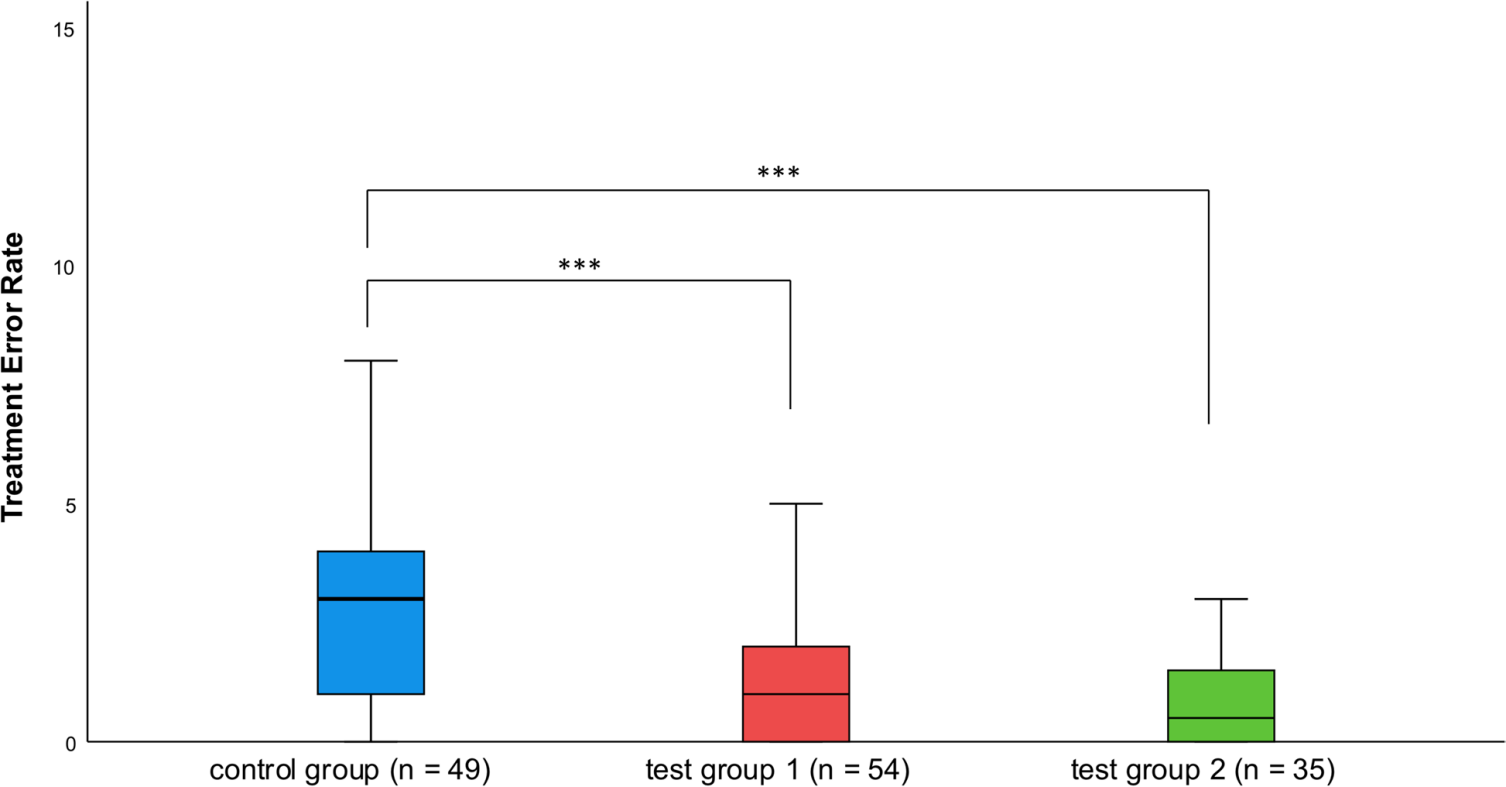
Graphic of the treatment errors registered during students’ first root canal treatments on real patients during their first clinical endodontic course. The scale of the *y*-axis ranges from 0 to 20. Medians and quartiles represent the results of the three groups**.** Asterisks mark the level of significance (* for *P* < 0.05; *** for *P* < 0.001)

The number of treatment errors significantly decreased from 3.2 ± 2.9 in the control group to 1.2 ± 1.3 in test group 1 (*P* < 0.001). The number of treatment errors did not significantly differ between test group 1 and test group 2 (0.8 ± 0.9); however, it significantly differed between test group 2 and the control group (*P* < 0.001; Fig. 4).

The treatment errors were allocated to the already mentioned stages to enable a clear presentation. There were no significant differences between the control group and test group 1 regarding errors of isolation and opening of the cavity (stage 1). Moreover, no significant differences in determining the working length (stage 2) were observed. For root canal instrumentation and irrigation (stage 3), differences between the groups were significant on several points. During root canal preparation, periapical tissue damage was significantly lower in test group 1 than in the control group (Item 6; *P* < 0.05). They were less likely to show incorrect root canal preparation (Item 8; *P* < 0.01), resulting in elbow zips, blockings of root canals, and lateral perforation of canal walls. Errors regarding the irrigation process were observed in the control group but not observed in the test group (Item 9; *P* < 0.05). Errors regarding obturation and restoration were summarized as stage 4. Significantly fewer radiographs with master cones perforating the apex were recorded in test group 1 (Item 10; *P* < 0.001). When assessing the root canal filling, in particular, fewer inhomogeneous (Item 12; *P* < 0.05) and discontinuous (Item 13; *P* < 0.05) root fillings occurred. Post-operative radiographs showed significantly more insufficiently cleaned cavities (Item 16; *P* < 0.01). Additional errors such as the wrong application of permanent post-endodontic filling or the wrong usage of sealer components were less frequently observed in test group 1 than in the control group (Item 20; *P* < 0.01). There were no significant differences between test groups 1 and 2 regarding the error rate, but there were some non-significant differences as the error rate was higher for a few items.

### Evaluation of the endodontic multimedia application

A total of 89 students were eligible to participate in evaluating the endodontic multimedia application using the SUS and a 5-point Likert scale containing 14 questions. Five students did not complete the questionnaire, leading to a response rate of 94%. The reliability of the Likert scale was assessed using Cronbach’s alpha, which was 0.829 and thus indicated high internal consistency [45]. The endodontic multimedia learning application received fairly good ratings from students in both test groups (4.3 ± 0.6; Likert scale: 1–5, Table 2).

**Table 2:** Assessment of the learning application regarding students’ satisfaction using a 5-point Likert scale questionnaire (test groups 1 and 2; *n* = 84 students)

Item	5	4	3	2	1
I would recommend the learning application to someone	51.2% (43)	36.9% (31)	10.7% (9)	0% (0)	0% (0)
I find the tooth illustrations appealing	38.1% (32)	45.2% (38)	13.1% (11)	0% (0)	0% (0)
I find the animation of the tooth illustrations appealing	39.1% (33)	47.6% (40)	10.7% (9)	0% (0)	0% (0)
I find the structure of the learning application useful	46.4% (39)	41.7% (35)	8.3% (7)	2.4% (2)	0% (0)
I find the presentation of the different tooth types superfluous	1.2% (1)	3.6% (3)	10.7% (9)	33.3% (28)	47.6% (40)
I find videos on artificial teeth helpful for my learning process	56.0% (47)	28.6% (24)	11.9% (10)	2.4% (2)	0% (0)
I find videos on endodontic patient treatments helpful for my learning process	71.4% (60)	21.4% (18)	4.8% (4)	0% (0)	0% (0)
The videos convey all the necessary aspects of endodontic treatment	28.6% (24)	57.1% (48)	11.9% (10)	1.2% (1)	0% (0)
Through the videos, I can understand the endodontic treatment	38.1% (32)	52.4% (44)	8.3% (7)	0% (0)	0% (0)
I find the jump marks of the videos superfluous	1.2% (1)	1.2% (1)	16.7% (14)	35.7% (30)	44.0% (37)
I find the length of the videos appropriate	41.7% (35)	42.9% (36)	14.3% (12)	0% (0)	0% (0)
I had difficulties operating the player	6.0% (5)	4.8% (4)	4.8% (4)	16.7% (14)	66.7% (56)
I find the speed function of the player helpful	25.0% (21)	23.8% (20)	38.1% (32)	10.7% (9)	1.2% (1)

5 = applies strongly, 4 = applies predominantly, 3 = undecided, 2 = does not apply predominantly, 1 = does not apply

Both test groups assigned the application an excellent rating on the SUS (SUS score: 81.4, Table 3).

**Table 3:** Assessment of the endodontic e-learning application on the SUS (test groups 1 and 2; *n* = 84 students)

Item	5	4	3	2	1
I can imagine using the learning application regularly	38.1% (32)	44.0% (37)	8.3% (7)	7.1% (6)	1.2% (1)
The learning application is unnecessarily complex	0% (0)	1.2% (1)	10.7% (9)	40.5% (34)	46.4% (39)
The learning application is easy to use	44.0% (37)	38.1% (32)	11.9% (10)	4.8% (4)	0% (0)
I would need technical support to use the learning application	0% (0)	3.6% (3)	15.5% (13)	25.0% (21)	54.8% (46)
The different functions of the learning application are well-integrated	32.1% (27)	47.6% (40)	19.0% (16)	0% (0)	0% (0)
The learning application contains many inconsistencies	0% (0)	2.4% (2)	15.5% (13)	52.4% (44)	28.6% (24)
The learning application is quick to master	56.0% (47)	25.0% (21)	15.5% (13)	2.4% (2)	0% (0)
The operation is very cumbersome	0% (0)	1.2% (1)	8.3% (7)	28.6% (24)	60.7% (51)
I felt very confident using the learning application	41.7% (35)	38.1% (32)	16.7% (14)	2.4% (2)	0% (0)
I had to learn many things before I could work with the learning application	0% (0)	3.6% (3)	15.5 (13)	29.8% (25)	50.0%(42)

5 = applies strongly, 4 = applies predominantly, 3 = undecided, 2 = does not apply predominantly, 1 = does not apply

### Evaluation of the COVID-19-specific survey

A questionnaire based on a 7-point Likert-type scale with 10 COVID-19-specific questions was distributed among the students in test group 2. The survey was completed by 35 participants, corresponding to a response rate of 100%.

**Fig. 5:**
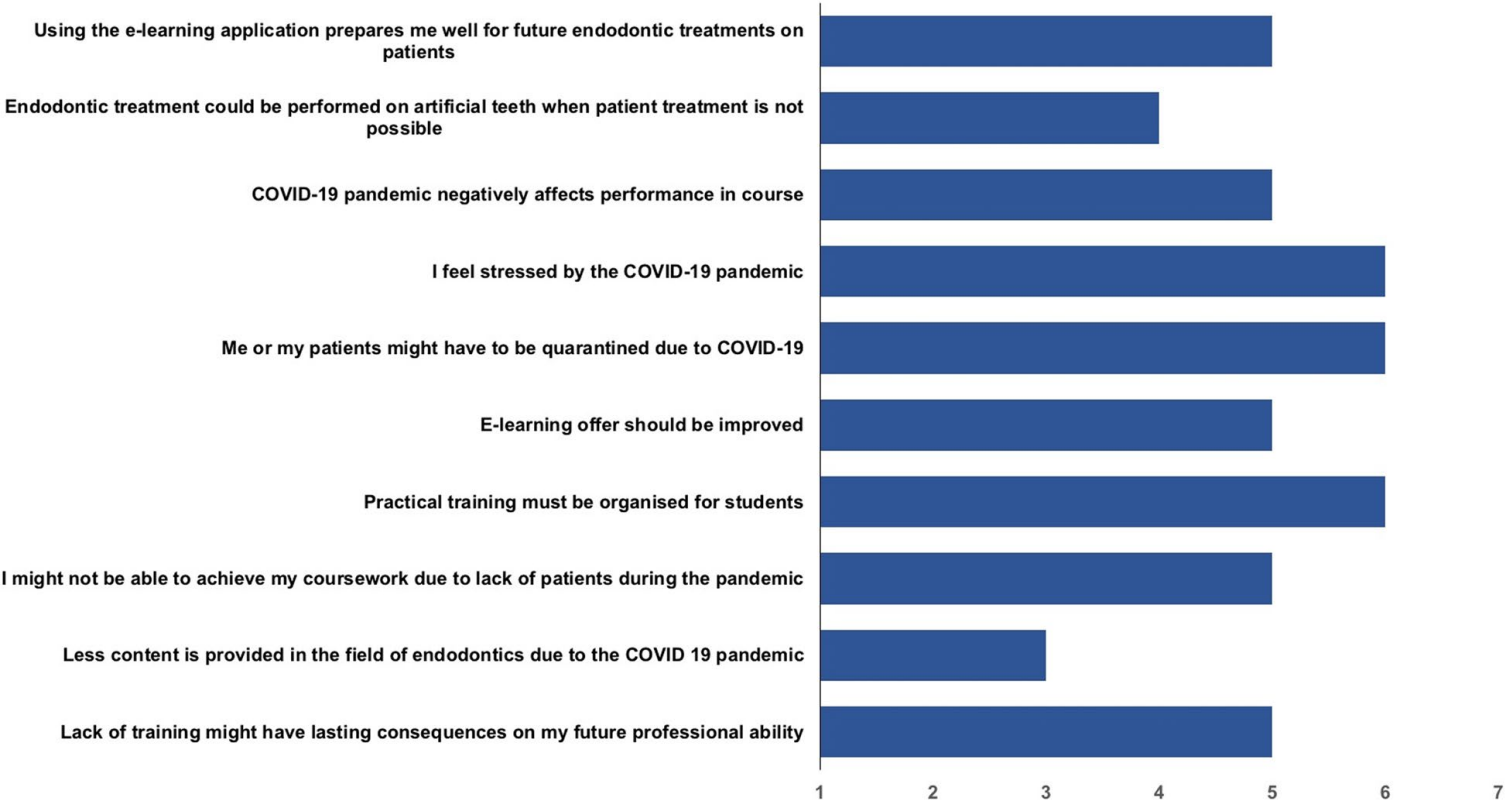
The bar chart presents the results of the Covid-19 survey containing 10 7-point Likert scale questions (1 = fully disagree to 7 = fully agree with 4 as a neutral response option). The *X*-axis shows the rating from 1 to 7, whereas the bars mark the median for each question

For all questions, the scale ranged from 1 (fully disagree) to 7 (fully agree), with 4 as a neutral rating option. A median score for each question was calculated since the Likert-type scale data were treated as ordinal data (Fig. 5).

## Discussion

E-learning has gained more importance recently in medical and dental education [46–50]. The availability of digital infrastructure and appropriate training for students and tutors are challenges associated with e-learning [51, 52]; nevertheless, digital learning has become an indispensable part of modern teaching. In 2014, Brumini et al. demonstrated dental students’ positive attitude toward e-learning [53]. As a significant benefit, e-learning applications enable students to learn at their own pace, repeatedly accessing course content and practice material as needed [35]. Various studies have indicated that e-learning enhances dental education [14, 54, 55]. As a mixture of e-learning and conventional learning, the blended learning approach has been successfully integrated into medical and dental teaching and has proven to increase students’ performance. Although some students are critical of the increased time spent at home using multimedia applications instead of face-to-face lectures [56], e-learning offers the advantage of viewing the study content repeatedly [57].

Students have preferentially adopted blended learning approaches [16, 58, 59], which is unsurprising since present generations of students are quite familiar with media and use it regularly for learning purposes [16]. Similar findings have been described in other studies, where explanatory videos have received broad approval from learners [12, 60, 61]. Excellent ratings for user-friendliness and design and technical implementation affirm the quality of the application. Good acceptance among students is crucial to adopt a learning application successfully [17].

Our results support the positive effects of e-learning. The first null hypothesis is rejected due to a significant improvement in the performance of test groups 1 and 2 compared to the control group. The e-learning application was intended to prepare students for the subject of endodontics before the first clinical endodontic course, stimulating self-learning in students as expected in flipped classroom approaches [14, 35]. For example, in 2020, Singal et al. stated that the ability to learn on one’s own is of great importance [29], which is especially true during the current COVID-19 pandemic period, with the wide use of distance learning [62].

Successfully performing root canal treatments is a highly technical procedure that requires strict adherence to procedural processes [63]. Previous research has suggested that students are better prepared to perform procedural processes through blended learning than through classical teaching methods alone [64]. Our findings show improved performances for students who used the e-learning application. Previous studies have shown that blended learning approaches positively affect students’ performances [17, 65]. When evaluating the e-learning application, students in our study conveyed its positive effect on their learning process.

Digitalization in university teaching, in general, is more topical than ever during the pandemic [21, 30, 66, 67]. The implementation of online learning seems to affect students’ motivation positively [68]. Therefore, during the pandemic, this approach, combined with converting lectures and seminars to online presentations, may have helped compensate for the lack of physical interaction in the theoretical part of our course. Apart from hands-on training on extracted and artificial teeth, only patient treatments continued to be performed in person. There was no significant difference between the test groups. Thus, the 2^nd^ null hypothesis is rejected since conducting lectures and seminars as online events had no negative effect on the result of students’ first root canal treatment on a real patient. Due to high infection rates, physical attendance at lectures was temporarily restricted, so alternatives had to be found to ensure endodontic teaching, similar to other disciplines [20, 21]. Compared with test group 1, test group 2 was significantly less successful in determining the correct working length based on the preoperative radiographs, whereas they significantly more often established accurate root canal lengths. As the students of all groups had guidance by an experienced specialist in endodontics especially during their first treatment, a more frequent use of the video instructions could be a possible explanation for this finding. Determining the correct working lengths highly depends on experience, and the video instructions might not have been able to improve this treatment step as well as others.

Students in our department reported enjoying attending clinical endodontic courses involving patient treatments despite the pandemic and supported our efforts to continue teaching. Continuing endodontic teaching with a real patient during the pandemic was a significant challenge, and a high level of additional infection control measures was necessary to minimize the risk of infection for patients, students, and staff [27, 32]. Psychologically, mental stress and concerns about the pandemic made it challenging for students to focus on their studies [32]. Although the COVID-19 pandemic caused additional student stress, and the loss of face-to-face teaching was harsh [68], our results imply that a well-coordinated e-learning application combined with online teaching and practice on artificial teeth might have contributed to the maintenance of teaching endodontics during the pandemic, enhancing and modernizing traditional endodontic teaching methods.

As the test group 2 achieved the best results for their root canal treatments, our findings may indicate the possibility of replacing teachers with video instructions. However, there are studies that shed light on the disadvantages of online teaching without any in-person events. The delivery of online content can be differentiated as asynchronous (e.g., pre-recorded lectures, video instructions) and synchronous (e.g., live lectures, in-person seminars) [69]. Students seemed to prefer a mixture of both these forms [70]. However, they were not in favor of completely replacing face-to-face teaching with online teaching [71] as they noted a lack of communication within the group and with the lecturers [72–75]. According to a recent study, students claimed online learning to be less effective balancing theory and practice as well as in building of practical skills [76]. Previous studies have found that blended learning approaches are preferentially adopted by students [16, 58, 59]. In a randomized controlled trial, the authors could demonstrate the superiority of blended learning approaches compared to face-to-face and online approaches for the technical sensitive procedure of local anesthesia [77]. This finding is in line with studies reporting on the superiority of flipped classroom approaches [14, 58, 78]. In any event, professional staff as well as offline lessons remain crucial for successful teaching and cannot be completely replaced [76].

According to our findings, the e-learning application enabled students to achieve more effective practical exercises on artificial teeth resulting in a better understanding of the treatment steps. Several studies have investigated using artificial teeth as substitutes for natural teeth in preclinical courses [7, 10, 40, 79, 80], while others have compared different kinds of artificial teeth [81]. In the present study, artificial teeth were utilized as additional training aids in endodontic pre-course settings before entering the first clinical course, combined with an e-learning tool. Significant differences in endodontic performance between inexperienced students and experienced operators have been found [82–85]. These studies have identified the importance of students practicing manual skills before entering clinical courses. For example, a recent study considered the treatment of 6 simulated root canals reasonable to attain an adequate level of competence [86], indicating the urgent need for these training aids. However, the exclusive use of an e-learning tool without any training on artificial teeth may not have yielded these positive results.

The use of artificial teeth provides a safe environment for students, so they can practice without the risk of harming the patient when treatment errors occur. Nevertheless, training on real patients is irreplaceable for gaining endodontic skills [87]. Possible reasons for this benefit include adequate communication with the patient, possible pain development, and restricted access to the treatment site due to the patient’s anatomy. The transition from preclinical knowledge to real clinical cases is highly stressful for students [88–92], which, according to our findings, has been especially true during the COVID-19 pandemic. Therefore, it is crucial to overcome the theory–practice gap that often prevents students from successfully transferring their knowledge to a clinical situation [89, 93]. Eventually, all endodontic teaching methods aim to prepare students as effectively and realistically as possible to perform root canal treatments on actual human beings successfully.

## Limitations

Our study design did not involve randomization, and there were no parallel performances of the three groups. All students in a particular semester were assigned to the same group to require no exclusion of additional learning content for one group of students within a semester. Students in the same semester could not be divided into different groups as there would have been unequal conditions for the students to pass their examinations. Furthermore, none of the students wanted to waive the opportunity for additional educational content voluntarily. Therefore, the results of the present study might have been influenced by time-dependent selection bias since a variation might have existed between the student groups and patients who were endodontically treated. Moreover, the study design did not distinguish between the effect of the e-learning application and the use of tooth models. However, both methods are effective in dental teaching [17, 77–79, 94].

Future studies should analyze the effect of the learning application more intensively, illuminating the difference in students’ performances between the use of artificial teeth and the use of artificial teeth with the learning application compared to traditional exercises on extracted teeth. Ideally, blind randomized trials with parallel performances should be used. The limitation of the small number of students per semester in dental schools could be addressed by performing multicenter studies to obtain larger cohort sizes.

## Conclusion

The multimedia e-learning application improved students’ performance in the first root canal treatment on real patients. The individualized learning pace of the students and an illustration of the treatment steps enhanced the students’ theoretical and practical knowledge of endodontic treatments. Thus, practical exercise is immensely significant for students to achieve expertise and practice and gain self-confidence in complex endodontic treatments. Indeed, e-learning plays a supportive role in this process. During the COVID-19 pandemic, the e-learning application, combined with online lectures, appeared to maintain the quality of students’ endodontic treatments. However, performing root canal treatments on real patients remains crucial to provide knowledge about managing stressful situations and patients. Therefore, a possible further research approach could be evaluating students’ stress levels during patient treatments and analyzing how the video instructions helped the students with problem-solving.

## Supplementary Information

**Additional file 1.** 

## Data Availability

All data are available from the corresponding author upon reasonable request.

## Abbreviations

COVID-19 pandemic: Coronavirus disease pandemic
DRSK RCT: Name of the tooth model presented in this study
