# Supplementary material for: Effect of an endodontic e-learning application on students’ performance during their first root canal treatment on real patients: a pilot study
Source: BMC Med Educ. 2022 May 23;22:394. doi: 10.1186/s12909-022-03463-y (PMC9124745; doi:10.1186/s12909-022-03463-y)
Supplement: Supplementary file 1 — Additional file 1. [file 12909_2022_3463_MOESM1_ESM.docx]

**Bullet sheet for success rate of endodontic treatments.**

**Stage 1 Isolation and preparing of access cavity and visualization of the orifice**

Item 1 Access cavity is orientated according to the tooth axis.

Item 2 Expansion of the access cavity substance-preserving.

Item 3 Access cavity allows instrumentation of the root canals without excessive bending (straight line access).

Item 4 Pulp chamber roof is completely removed.

Item 5 Accurate preparation of the orifices in relation to the root canal anatomy.

**Stage 2 Determination and control of working length**

Item 6 Root canal has accurate working length in relation to radiographic image.

Item 7 Marking of the working length corresponds to the reference point.

Item 8 Radiographic visibility of the reference point, the apex and the device used for length measurement.

Item 9 Working length is correctly determined.

Item 10 Correct documentation of the working length and the reference point.

**Stage 3 Canal instrumentation and irrigation**

Item 11 Master file is selected according to the original diameter of the root canal.

Item 12 Master cone has tug back.

Item 13 Reference point and master cone are clearly visible on radiographic image.

Item 14 Master cone extends 0.5 – 1.0 mm before radiographic apex.

**Stage 4 Obturation and restoration**

Item 15 Gutta-percha is clearly shortened to crestal level.

Item 16 Cavity walls are free of sealer or gutta-percha.

Item 17 Root filling extends 0.5 – 1.0 mm before radiographic apex.

Item 18 Root filling is homogeneous.

Item 19 Root filling is compacted without air entrapments/ bubbles.

Item 20 Root filling is continuously conical.

Item 21 Root canal preparation is appropriate and sufficient regarding the original shape of the root canal.

**Bullet sheet for error rate of endodontic treatments.**

**Stage 1 Isolation and preparing of access cavity and visualization of the orifice**

Item 1 Rubber dam is missing or incorrectly placed.

Item 2 Grinding/ perforation of the pulp chamber floor.

**Stage 2 Determination and control of working length**

Item 3 Device for length measurement perforates the apex.

**Stage 3 Canal instrumentation and irrigation**

Item 4 Lateral perforation of the root canal.

Item 5 Instrument fractures inside the root canal.

Item 6 Injury to periapical tissue.

Item 7 Incomplete preparation of the root canal.

Item 8 Errors in root canal preparation (step/ elbow zip/ blocking/ …).

Item 9 Error in irrigation of the root canal.

Item 10 Master cone perforates the apex.

Item 11 Master cone does not match to the reference point.

**Stage 4 Obturation and restoration**

Item 12 Root filling is not homogeneous.

Item 13 Root filling does not have continuous conicity.

Item 14 Root filling extends more than 1.0 mm before radiographic apex.

Item 15 Root canal is overfilled.

Item 16 Cavity walls are not clean of sealer or gutta-percha.

Item 17 Endodontically treated tooth breaks off during/ after treatment.

Item 18 Recurrence of complaints (flare up/ swelling/ periapical lesion/ …).

Item 19 Parts of the root canal remain unfilled.

Item 20 Other.
